# Total Factor Productivity and High-Quality Economic Development: A Theoretical and Empirical Analysis of the Yangtze River Economic Belt, China

**DOI:** 10.3390/ijerph19052783

**Published:** 2022-02-27

**Authors:** Shaolong Zeng, Xianfan Shu, Wenxian Ye

**Affiliations:** 1 School of Economics, Hangzhou Normal University, Hangzhou 311121, China; shaolongzeng@hznu.edu.cn; 2 School of Management, Shanxi Institute of International Trade & Commerce, Xi’an 712046, China; yewenxian@csiic.edu.cn

**Keywords:** Yangtze River Economic Belt, total factor productivity, high-quality development, influencing factors, business environment

## Abstract

This paper focuses on the total factor productivity (TFP) and high-quality economic development in China by examining 11 Chinese provinces and cities in the Yangtze River Economic Belt from 2007 to 2018. We use the Solow residual method to calculate the TFP growth rate of the 11 provinces and cities. Based on the panel data, we have analyzed the influencing factors of TFP theoretically and empirically from the overall region and upstream region, and midstream region and downstream region, respectively. The regression results show that: (1) The whole characteristics generally show the TFP growth trend of the upstream region, midstream region and downstream region are consistent with that of the overall region, and the growth rate of TFP slows down gradually. Meanwhile the differences in TFP growth between the upstream region, midstream region and downstream region show an increase at first and then a decrease. (2) Regarding the influencing factors, there are differences in the direction and extent of the impact of each factor such as the level of openness, R&D investment, industrial structure, government expenditure and human capital on the TFP of the overall region, upstream region, midstream region and downstream region. (3) Based on the results of the theoretical and empirical analysis, we have proposed a series of measures for the sustainable high-quality development of the Yangtze River Economic Belt.

## 1. Introduction

High-quality economic development is an important goal for China in the new era, and total factor productivity (TFP) is an important measure of high-quality economic development. In the report of the 19th National Congress of the Communist Party of China (CPC) and the 2nd, 3rd, 4th, 5th and 6th Plenary Session of the 19th CPC Central Committee, it was emphasized that ‘We must put quality first and give priority to performance. We should pursue supply-side structural reform as our main task, and work hard for better quality, higher efficiency, and more robust drivers of economic growth through reform. We need to raise total factor productivity …’ [1]. All provinces and cities in China regard effectively improving TFP and achieving high-quality economic development as their top priority. China also emphasizes that innovation is the fundamental driving force for further deepening reforms, further opening up the economy, and building new advantages in global competition. Therefore, how to improve TFP through innovation is the key to cracking the driving force of China’s sustainable economic development, and it is also a problem that all economic entities urgently need to solve in the construction of a market economy. Zhang and Huang found that it is an urgent challenge to coordinate technological innovation, resource consumption, environmental quality and high-quality industrial development in China [2]. Zhang et al. thought that improving factor allocation can accelerate the TFP and promote the high-quality development of China’s economy [3].

The Yangtze River Economic Belt plays a pivotal role in China’s economy and has a first-development advantage, showing a complete range of industries, abundant resources and a solid development foundation. At the same time, there are also some deficiencies, such as the accumulation of a large number of environment-polluting industries and traditional manufacturing industries, which are in a critical period of economic transformation, and upgrading and overall improvement of TFP. The overall development level of the Yangtze River Economic Belt is relatively high. However, there is not only the Yangtze River Delta region with a developed economy and outstanding advantages, but also Sichuan, Yunnan and Guizhou regions which are relatively underdeveloped [4]. There are large differences between provinces and cities in terms of industrial structure, opening-up, technological innovation, human capital and local policies. These differences are ultimately reflected in the differences in TFP between provinces and cities, which are important factors for the current uneven and insufficient economic development in China.

In December 2019, the Central Committee of CPC and the State Council issued the ‘Outline of the Yangtze River Delta Regional Integrated Development Plan’, marking the official rise of the integrated development of the Yangtze River Delta into a national strategy, and the diffusion effect of the Yangtze River Delta region will drive the development of the Yangtze River Economic Belt to achieve coordinated regional development [5]. In April 2018, General Secretary Xi Jinping clearly stated to ‘correctly grasp the five major relationships and promote the high-quality development of the Yangtze River Economic Belt’ at the Symposium on Deepening the Development of the Yangtze River Economic Belt [6]. In fact, early in 2017, five departments including the Ministry of Industry and Information Technology jointly issued the “Guidance on Strengthening Green Development in the Industrial Sector of the Yangtze River Economic Belt” (Ministry of Industry and Information Technology (2017) No. 178), specifying that a green manufacturing system will be initially established in the Yangtze River Economic Belt by 2020. It can be seen that the Yangtze River Economic Belt is bound to be China’s green economic development belt, a high-quality economic development belt, and a demonstration belt of the market economy with Chinese characteristics. Therefore, optimizing the industrial structure of the 11 provinces and cities along the Yangtze River and improving the TFP of the Yangtze River Economic Belt as a whole is the key to taking the lead.

It is an important connotation of high-quality economic development to improve TFP. Effective measurement of TFP is a prerequisite for an objective and comprehensive understanding of the quality level of economic development. At the same time, there are differences in research perspectives and considerations in the analysis of factors affecting TFP. We take 11 provinces and cities in the Yangtze River Economic Belt as the research object, measure TFP and analyze the current situation and differences of TFP for the overall region, upstream region, midstream region and downstream region. The overall region includes all the 11 provinces and cities in the Yangtze River Economic Belt, which are Shanghai, Jiangsu, Zhejiang, Anhui, Hubei, Hunan, Jiangxi, Sichuan, Yunnan, Guizhou and Chongqing. The downstream region includes Shanghai, Jiangsu, Zhejiang and Anhui. The midstream region includes Hubei, Hunan and Jiangxi. The upstream region includes Sichuan, Yunnan, Guizhou and Chongqing. Then, we empirically examine the influencing factors of TFP, including the level of openness, research and development (R&D) investment, industrial structure, government expenditure and human capital on TFP in the overall region, upstream region, midstream region and downstream region. Based on these, we have proposed a series of measures for the sustainable high-quality development of the Yangtze River Economic Belt to improve the TFP of the Yangtze River Economic Belt from an overall and regional perspective, narrow regional differences, and achieve regional coordination and high-quality development.

The rest of the paper is structured as follows: Section 2 provides a literature review, Section 3 a theoretical analysis, Section 4 data sources and empirical models, and Section 5 gives empirical results and robustness tests. Section 6 concludes with policy recommendations. The abbreviations of key terms are showed in Appendix A Table A1.

## 2. Literature Review

TFP refers to the ratio of the total output of an economic system to the real input of all factors in production. It is often used to analyze the source and driving force of economic growth. Therefore, for China to achieve industrial transformation and upgrading and high-quality economic development, the key is to comprehensively improve TFP. The relevant research on TFP mainly involves the relationship between TFP and economic growth, and the measurement, the differences and the influencing factors of TFP.

### 2.1. The Relationship between TFP and Economic Growth

With the improvement of the level of economic development, people pay more attention to TFP and regard the improvement of TFP as an important symbol of high-quality economic development. Zhang concluded that TFP can significantly promote high-quality economic development, but it must be based on correct government intervention [7]. Liu et al. proved through empirical analysis that TFP can significantly affect economic growth and widen the economic gap in China, but its role is relatively small [8]. Tu found that TFP is an important factor affecting regional economic growth, and the key is technological advancement [9]. With the deepening of supply-side structural reform, TFP has become a new driving force for the coordinated development of regional economies.

### 2.2. Calculation of TFP

Solow discussed the relationship between production function and productivity and obtained the Solow residual value based on the Cobb–Douglas production function, which is called the Solow residual method for measuring TFP [10]. This method is widely used by researchers due to its simplicity in measurement and calculation. However, there is also a lot of controversy. The main point of questioning is that the scale benefits are assumed to be constant and Hicks-neutral in the calculation. At the same time, in application, based on the differences in index selection and calculation methods, even the calculation results of TFP in the same region and the same period will inevitably be different, but their growth trends or rates of change are roughly similar. The calculation China’s TFP mainly focuses on three levels as below.

The first is the regional level, that is, the measurement and analysis of the TFP of the whole country or relevant provinces and cities. Guo and Jia estimated the TFP growth rate of China from 1979 to 2004 using the Solow residual method, the latent variable method and the potential output method [11]. Yang used the Solow residual method and the DEA-Malmquist index method to calculate the TFP of China’s provinces and cities from 1986 to 2014 [12]. Luo used the Solow residual method to estimate the elasticity of capital stock and labor to output in Beijing-Tianjin-Hebei from 2006 to 2016, so as to measure TFP and its index [13].

The second is the industry level, which is to measure and analyze the TFP of different industries. Lu and Lian estimated the TFP of major industrial enterprises in China [14]. Yang analyzed the dynamic changes of China’s manufacturing TFP and concluded that there are great differences in the TFP of enterprises with different ownerships [15].

The third is the combination of regions and industries, which take into account not only regional differences, but also industry differences. The Research Group of Measurement and Comparison of Total Factor Productivity in the Yangtze River Economic Zone used the Solow residual method and the DEA-Malmquist index method to measure the TFP of the overall region, upstream region, midstream region and downstream region from 2002 to 2016, and the industry is divided into industrial and service industries [16].

### 2.3. Regional TFP Differences

Due to the differences in the stage of development, economic development mode and resource endowment between different countries or regions, and different provinces and cities, TFP shows great differences. Some studies have conducted in-depth research on the differences in TFP in different regions. At the national level, Du used empirical analysis to conclude that there are significant regional differences in the growth of TFP in China, and the growth of TFP in the eastern region is faster than that in the central and western regions [17]. Yang found the same result as the growth rate of TFP in the eastern region is higher than that in the central and western regions [12]. At the regional level, Chen et al. analyzed the regional differences and influencing factors of TFP growth in the Yangtze River Economic Belt empirically, and the results showed that regional differences in TFP growth continued to increase [18]. In fact, the difference in the level of economic development between countries and between different regions of a country is the best evidence for the difference in TFP and its growth rate.

### 2.4. Factors Affecting TFP

According to the Cobb–Douglas production function, TFP refers to the sum of various factors other than capital and labor that contribute to an increase in output [19]. TFP is not a specific factor, nor a factor that can be directly measured like capital, but a general concept which is the ‘product’ of various factors that are difficult to measure specifically. Therefore, the factors affecting TFP have neither a fixed range nor a standard mechanism, but diversity and uncertainty. This better explains the large differences in the research on related factors affecting TFP.

#### 2.4.1. Level of Openness

The market economy is opening, the level of which is a key factor that has a significant effect on the TFP. Wei conducted empirical research and found that international trade and human capital are positively related to TFP [20]. Li and Tang empirically tested that international trade and foreign direct investment (FDI) were also positively related to TFP [21]. Jahanger found that the export capacity of the eastern provinces in China of FDI significantly promote high-quality economic development in the region; the technical level of the central provinces of FDI significantly promotes high-quality economic development in the region, but then the actual size of the FDI has a significant inhibitory effect on central provinces [22].

#### 2.4.2. R&D and Technological Innovation

Sometimes, researchers take technology and TFP equally. Yang concluded that China’s TFP is mainly driven by technological advancement [12]. The Research Group of Measurement and Comparison of Total Factor Productivity in the Yangtze River Economic Zone found that technological advancement significantly boosted the growth of TFP [16]. Chen et al. studied the reasons for the differences in TFP inside and outside the Yangtze River Economic Belt and concluded that a higher level of technological and financial advancement can promote the growth of TFP, while industrial structure and FDI will inhibit the growth of TFP [18].

Under the strategy of innovation-driven development, how to improve TFP through innovation is the key to promoting high-quality economic development. Wang et al. studied the effect of R&D investment on TFP, which shows that R&D investment has a significant role in promoting TFP, and there is a U-curve relationship between them [23]. Philipp et al. analyzed whether different R&D activities show a positive influence on TFP for firms of different ownership types and across two time periods. Overall, strong increases in the size of patent stocks are related to a decreasingly positive or even vanishing influence on TFP [24]. Zhang et al. found that on the whole, TFP is increasing annually, with technical progress being the main factor that affects the total efficiency [25].

#### 2.4.3. Other Factors

There are some other factors, such as industrial structure, institutional factor and human capital. On industrial structure, Yu et al. concluded that the upgrading of China’s industrial structure has effectively promoted the growth of TFP [26]. The institutional factor mostly means the government’s influence on the market economy. Wang et al. suggested that it is necessary to give full attention to the synergy between FDI and fiscal expenditure and formulate regionally targeted policies to improve GTFP and promote high-quality development in China [27]. Song et al. found that the expenditure decentralization and the fiscal expenditure competition among different areas are conducive to improving the local area’s GTFP [28]. Li et al. thought the institutional influences on the input–output of the high-tech industry are very clear and positive, and its influence on the TFP is the greatest [29]. On human capital, Zhao and Yuan found that labor supply loss, counter urbanization and human capital disruption are the three major transmission channels through which haze pollution affects the quality of China’s economic development [30]. Li et al. found the effects of the fixed asset investments and labor inputs in the coal resource-rich regions of Midwestern China on the TFP of the enterprises were minimal [31].

To sum up, the influencing factors of TFP mainly include the level of openness, R&D investment, industrial structure, government expenditure, human capital, etc. Therefore, based on the research of related literature, we use the panel data of 11 provinces and cities in the Yangtze River Economic Belt from 2007 to 2018, use the Solow residual method to calculate the TFP, and conduct an empirical analysis of its influencing factors. It has a certain theoretical significance and practical reference value for the high-quality economic development of the Yangtze River Economic Belt.

## 3. Theoretical Mechanism Analysis

Based on the relevant literature review, the influencing factors of TFP in the Yangtze River Economic Belt mainly include the following: level of openness, R&D investment, industrial structure, government expenditure and human capital. The mechanism of each influencing factor is as follows.

### 3.1. Level of Openness

The level of openness is mainly reflected in two aspects, international trade and FDI. The indicators are measured by the degree of dependence on international trade and the degree of dependence on FDI. The higher degree of dependence indicates the relatively higher level of openness. Expanding opening-up is to change the innovation level of enterprises through market competition and economies of scale, thereby increasing TFP. The competition mechanism is the key to international trade. Both import and export enterprises face challenges from global markets. Only enterprises with competitive advantages can survive in the global market competition and obtain greater profit margins. Therefore, participating in international competition is conducive to promoting TFP.

On the other hand, economic globalization is based on the horizontal and vertical specialization of production, which promotes the internationalization of enterprises and the deepening of FDI. This process realizes economies of scale by optimizing and extending the industrial chain and expanding the value chain, thereby further promoting the overall increase in factor productivity. Therefore, a region with a higher level of openness has a higher TFP.

### 3.2. Technological Advancement

The core of the endogenous economic growth theory of technological advancement is the main driving force for sustained economic growth. The creation of human wealth is the process of material capital accumulation, and it is also the evolution of technological advancement. Achieve technological advancement in product research, development and innovation, and at the same time endogenize technological advancement, further change the product structure, promote the transformation of enterprises to technology-intensive, and ultimately achieve increasing returns on factor inputs. Therefore, the value-added process is actually the process of technological innovation. Of course, there is not intuitive quantitative indicators for measuring the technology level in the short term. R&D expenditures can be used instead of technology level, although some R&D does not necessarily bring benefits. We adopt the ratio of R&D expenditure to gross domestic product (GDP) as a surrogate variable for technological advancement. R&D promotes technological advancement through knowledge innovation and other forms, improves the efficiency of the use of production factors, and under the condition of inputting the same factors, more products will be produced, thereby promoting the growth of TFP. Therefore, regions with greater technological advancement have higher TFP.

### 3.3. Industrial Structure

Industrial structure is an important reflection of the level and quality of economic development. The more optimized the industrial structure, the higher the level and quality of its economic development. The optimization process of the industrial structure generally shows that the proportion of the primary and secondary industries in the overall economy gradually declines, and the proportion of the tertiary industry in the overall economy continues to rise. After the economy develops to a certain level, there is limited room for adjustment of the proportion of the primary industry. At this time, the optimization of the industrial structure is mainly to replace the secondary industry with the tertiary industry, or the proportion of the service industry is getting higher and higher. The proportion of the added value of the tertiary industry to GDP can be used to represent the level of industrial structure. The optimization process of industrial structure not only depends on the improvement of TFP, but also further promotes the growth of TFP. Therefore, the higher the proportion of the added value of the tertiary industry in GDP, the higher the TFP.

### 3.4. Institutional Factors

The institutional factor mainly reflects the degree of the government’s influence on the market economy. In neo-institutional economics, institutions are considered to be the most critical factor affecting economic growth. In fact, governments and markets have never been independent of each other, but there are differences in their mutual influence and role. The government can influence market economic activities through fiscal and monetary policies and foreign economic policies. The effectiveness of macroeconomic policy implementation depends on the cooperation of the ‘invisible hand’ of the market. Appropriate policies can stimulate market vitality and promote faster economic growth. For example, the government optimizes the business environment, improves infrastructure, and enhances service levels, all of which can provide conditions for enterprise cooperation and innovation activities, thereby promoting more efficient operation of enterprises. Therefore, active macro policies are conducive to improving TFP. We use the proportion of government general budget expenditure to GDP to represent institutional factors. Under the condition that other conditions remain unchanged, the higher the proportion of government general budget expenditure in GDP, the higher the TFP.

### 3.5. Human Capital

Human capital is an important resource endowment. In endogenous economic growth theory, human capital is also an important driving force for economic growth. In fact, human capital is an important support for technological advancement. The richer the human capital, the more conducive to promoting technological innovation. Human capital is formed by workers through formal education, training, on-the-job learning, etc., that is, the process of giving workers more skills or higher output efficiency. Workers acquire new knowledge and skills through learning and use them in production, transforming them into higher labor capital, thereby promoting the growth of TFP. Therefore, human capital and TFP are in the same direction. We use the total number of high school and higher education graduates to represent the human capital stock. Regions with higher human capital stock have higher TFP.

## 4. Data Sources and Empirical Models

### 4.1. Related Variables and Data Sources

#### 4.1.1. TFP Growth

Due to the difficulty in obtaining input–output-related data, we use the Solow residual method to measure the relative value of TFP in the Yangtze River Economic Belt and various provinces and cities, that is, TFP growth rate (*tfp*).

It is assumed that the production function of each province and city is the C-D production function *Y* = *AK^α^L^β^*, and satisfies the Hicks neutral condition: *α* + *β* = 1.

Therefore, taking the logarithm of both sides of the production function at different times gives Equation (1):

*ln*(*Y_t_*) = *ln*(*A_t_*) + *αln*(*K_t_*) + *βln*(*L_t_*) + *u_t_* (1)

Equation (1) further simplifies to Equation (2)

*ln*(*Y_t_*/*L_t_*) = *ln*(*A_t_*) + *αln*(*K_t_*/*L_t_*) + *u_t_* (2)

*Y_t_* is the actual output of the base period in 2005, *K_t_* is the capital stock, *L_t_* is the labor input, *α* is the capital output elasticity coefficient, *β* is the labor output elasticity coefficient and *u_t_* is the error term.

We can estimate *α* when it is assumed that the technological advancement factor *A_t_*, the capital stock *K_t_* and the labor input *L_t_* are independent of each other. We took the time series data of actual output, capital stock and labor input of the Yangtze River Economic Belt into Equation (2) and got *α* = 0.7. Equation (3) can be used to further calculate the *tfp*.

*tfp* = *y* − *αk* − (1 − *α*)*l* (3)

where *y* is the real GDP growth rate, *k* is the capital stock growth rate and *l* is the labor input growth rate. When calculating the capital stock, the perpetual inventory method is used, and the depreciation rate is estimated to be 9.6% based on Zhang et al. [32].

#### 4.1.2. Explanatory Variables

According to the above analysis, the factors affecting TFP include the level of openness, R&D investment, industrial structure, government expenditure and human capital. The meaning of each variable, measurement method and its relationship with TFP are shown in Table 1.

The level of openness includes international trade dependence (*TRADE*) and FDI dependence (*FDI*), which are expressed by the proportion of total import and export in GDP and the proportion of FDI in GDP of provinces and cities in the Yangtze River Economic Belt from 2007 to 2018. R&D investment (*TECH*) is expressed by R&D spending as a percentage of GDP. The industrial structure (*IS*) is expressed as the added value of the tertiary industry as a percentage of GDP. The government expenditure (*M*) is expressed as the government expenditure, that is, the general spending as a share of GDP. Human capital (*HC*) is expressed as total high school and higher education graduates.

The original data used in the calculation of above variables is from the statistical yearbooks of 11 provinces and cities in the Yangtze River Economic Belt from 2007 to 2019 and the website of the National Bureau of Statistics. The data of R&D expenditure comes from the Ministry of Science and Technology of China. The number of high school and higher education graduates is derived in part from educational yearbooks and macroeconomic databases, part of the data is not given directly, but is calculated from the relevant data in the statistical yearbook. Some data are missing or the data for the same period given in the yearbooks of the two years before and after are quite different. We had some technical issues in the calculation, for example, the data on the gross fixed capital formation of 11 provinces and cities in the Yangtze River Economic Belt in 2018 is missing. Considering the changing trend was stable, we replaced it with the average of the two years before and after. The number of employees in Shanghai in 2013 is inconsistent due to the large deviation in the statistical yearbook before and after 2013, and the number of people given in the two years before and after is inconsistent. In the calculation of employees, the data from 2012 to 2014 are replaced by the smoothed values of the two years before and after.

### 4.2. Model Selection and Construction

According to the theoretical analysis on the influencing factors of TFP, the constructed model is Equation (4):

*tfp_it_* = *β*_0_ + *β*_1_*lnTRADE_it_* + *β*_2_*lnFDI_it_* + *β*_3_*lnTECH_it_* + *β*_4_*lnIS_it_* + *β*_5_*lnM_it_* + *β*_6_*lnHC_it_* + *u_it_* (4)

In Equation (4), *i* represents different regions, *t* represents time, and *u_it_* is the error term. The *tfp* is the growth rate of TFP, so each influencing factor is in logarithmic form, that is, Equation (4) expresses the impact of the change rate of each influencing factor on the *tfp*.

### 4.3. Analysis of the Current Situation

**Figure 1:**
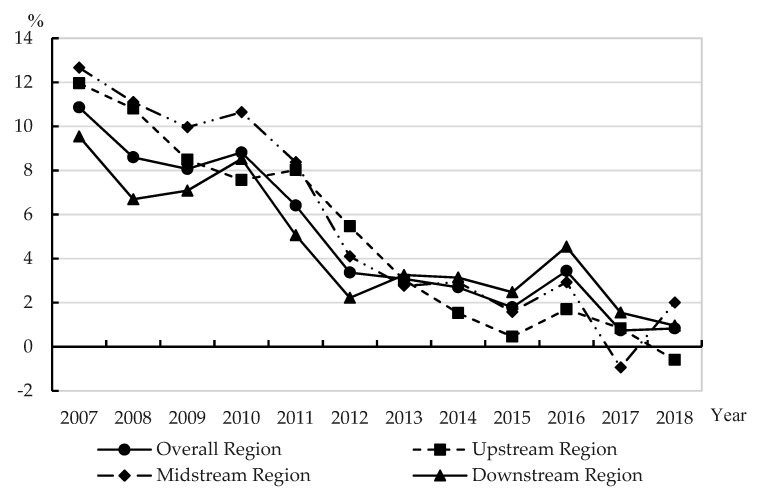
The TFP growth rate (*tfp*) of the Yangtze River Economic Belt from 2007 to 2018.

According to the relevant data of 11 provinces and cities in the Yangtze River Economic Belt from 2007 to 2018, the Solow residual method can be used to calculate the TFP growth rate (*tfp*) of the Yangtze River Economic Belt from the overall region, upstream region, midstream region and downstream region. See Table 2 and Figure 1.

Overall, the TFP growth rate (*tfp*) in the overall region of Yangtze River Economic Belt from 2007 to 2018 showed a downward trend, which was basically consistent with the change trend of GDP growth rate during this period. From the characteristics of the calculated data, the overall growth rate of capital was getting higher and higher, while the growth rate of labor was getting lower and lower, and the TFP growth rate (*tfp*) was decreasing after calculation. Theoretically, with the continuous improvement of the economic development foundation of the provinces and cities along the Yangtze River, the TFP gradually increased, but the pulling effect of various factors on TFP continued to decrease, so the TFP growth rate (*tfp*) declined.

From a region perspective, from 2007 to 2018, the TFP growth rate (*tfp*) of the upstream region, midstream region and downstream region was consistent with that of the overall region and showed a gradually decreasing trend. Since the capital elasticity coefficient *α* is uniformly 0.7 in this paper, the differences between regions have been reduced to a certain extent, and the actual differences should be larger. From the absolute value from 2007 to 2012, the TFP of the midstream region was the highest, followed by the upstream region, and that of the downstream region was the lowest. From the relative value from 2013 to 2017, the TFP growth rate (*tfp*) of the downstream region was the highest, followed by the midstream region, and that of the upstream region was the lowest. In 2018, the TFP growth rate (*tfp*) of the midstream region ranked first, while that of the upstream region was negative. The difference between the upstream region, midstream region and downstream region shows a trend of first expanding and then narrowing. Summary statistics of *tfp* in the four regions are shown in Table 3.

The dynamic changes of the TFP of different regions could be reasonably explained in terms of industrial transfer and upgrading and technological innovation. On the one hand, from the perspective of industrial transfer and upgrading, advanced industries such as high-tech industries first gathered in the downstream region. Then, due to industrial transfer, technological innovation factors were transferred between provinces, and some enterprises in the midstream region and upstream region achieved industrial transformation and upgrading. Meanwhile there is a phenomenon of overtaking the curve, crossing the order of industrial upgrading and directly entering into the high-tech industries. On the other hand, from the perspective of technical exchanges, with the deepening of opening up and the promotion of the construction of the ‘Belt and Road Initiative’, the open area has been promoted from the Yangtze River Delta region to the inland. With the improvement of infrastructure, the flow of talent has accelerated, making it much easier to have international technical exchanges and inflows and domestic technology exchanges between regions. Affected by the interaction of these factors, the growth rate of TFP tended to be stable, and the TFP gap between regions gradually narrowed.

## 5. Empirical Analysis and Results

### 5.1. Multiple Regression

The panel data of the overall region, upstream region, midstream region and downstream region in Yangtze River Economic Belt were used. We conducted a regression test based on empirical Equation (4). The regression results are shown in Table 4. 

By comparing the results of ordinary least squares, fixed effects and random effects, as results show by the Correlated Random Effects-Hausman test, the P values of the model of the overall region, upstream region and downstream region were 0.0028, 0.0000 and 0.0347; all are less than 0.05. So, a Fixed Effects Model was selected. While the *p* values of the model of the midstream region was 0.8323, which is greater than 0.05, so a Random Effects Model was used.

### 5.2. Testing

#### 5.2.1. Multicollinearity Test

According to the regression results, the absolute values of the correlation coefficients between variables are all less than 0.8. The VIF results of the overall region, upstream region, midstream region and downstream region model are 3.67, 5.24, 5.54 and 12.58, respectively. Except for the downstream region, the VIF values are all less than 10, indicating that there is not serious multicollinearity in the four models. We can use the original data for regression.

#### 5.2.2. Heteroskedasticity Test

Based on the White heteroskedasticity test, the *p* value of Obs*R-squared of the overall region, upstream region, midstream region and downstream region in Yangtze River Economic Belt are 0.0201, 0.2036, 0.1848 and 0.1129, respectively. Except for the overall region, the *p* values all passed the 5% significance test. This means that there is no heteroscedasticity in the upstream region, midstream region and downstream region models, but there is in the overall region model.

#### 5.2.3. Serial Correlation Test

We conducted a serial correlation test on the overall region, upstream region and downstream region models which were regressed with a Fixed Effects Model. The *p* values were 0.0000, 0.0143 and 0.0311, respectively, and passed the 5% significance test. The null hypothesis of no serial correlation was rejected, which means serial correlation existed.

The random effects serial correlation test was performed on the midstream region model, and the *p* value passed the 5% significance test too. The midstream region model also had serial correlation.

According to the test results, in order to solve the problem of the serial correlation of the four models and the heteroscedasticity of the overall region model, cluster robust standard errors were used to eliminate the serial correlation for the upstream region, midstream region and downstream region models. Additionally, the first-order lag TFP was added to the regression of the overall region model to obtain a robust statistic for the Fixed Effect Model.

### 5.3. Empirical Results

The regression results show the impact of the level of openness, R&D investment, industrial structure, government expenditure and human capital on the TFP of the overall region, upstream region, midstream region and downstream region in the Yangtze River Economic Belt. The analysis is as follows.

#### 5.3.1. International Trade

From the perspective of international trade, the *lnTRADE* coefficients of the overall region and upstream region of the Yangtze River Economic Belt are both negative, and the *lnTRADE* coefficients of the midstream region and downstream region are both positive. Statistically, the result of *lnTRADE* in all region models except the upstream region model passed the 1% significance test.

On the overall region, the result shows that the improvement of international trade level has a significant inhibitory effect on the growth of TFP, which is inconsistent with theoretical expectations. There is still a lot of room for the improvement of the level of openness of the Yangtze River Economic Belt, and the traditional quantitative effect of international trade should be transformed to the quality effect, so as to improve the TFP and promote high-quality economic development.

At the same time, from the regional point of view, there are obvious differences in the upstream region, midstream region and downstream region, and the coefficients of *lnTRADE* in the three regions’ models are −2.043, 1.869 and 5.252, respectively. It shows that Sichuan, Yunnan, Guizhou and Chongqing in upstream region have not fully integrated into the global production chain and value chain; Hubei, Hunan and Jiangxi in midstream region can keep up with the trend of economic globalization, but their advantages have not yet been exerted; Shanghai, Jiangsu, Zhejiang and Anhui in the in downstream region have strong international trade advantages and have entered the middle and high end of the industrial chain with both quality and quality.

#### 5.3.2. FDI

From the perspective of FDI, the *lnFDI* coefficients of the overall region, midstream region and downstream region of the Yangtze River Economic Belt are all positive, which are 0.272, 5.498 and 1.337, respectively. The *lnFDI* coefficient of the upstream region is −1.721. Additionally, the upstream region and midstream region passed the 1% significance test. Although the results deviate from the theoretical expectations, they are more in line with the actual situation. That is, the differences in developmental stages are ignored in the theoretical analysis.

In fact, the development stages of the upstream region, midstream region and downstream region are obviously different. The downstream region of the Yangtze River Economic Belt is the earliest open region and have gotten the highest level of development. This region is in the middle and high end of the global industrial chain, so the pulling effect of FDI is not so obvious as the other two regions. However, the upstream region and midstream region are relatively late in opening and are still in the stage of international industrial transfer. In particular, Hubei, Hunan and Jiangxi in the midstream region have initially formed a relatively complete industrial system, which can undertake advanced manufacturing and high-end industries at home and abroad. Therefore, FDI has the most significant role in promoting TFP in the midstream region than other regions.

#### 5.3.3. R&D Investment

R&D investment brings technological advancement and increases production efficiency. From the regression results, the *lnTECH* coefficients of the overall region and upstream region of the Yangtze River Economic Belt are 2.170 and 5.809, respectively, which are consistent with theoretical expectations. The *lnTECH* coefficients of the midstream region and downstream region are −7.209 and −15.99, respectively, which are inconsistent with theoretical expectations. Statistically, the result of *lnTECH* in all region models except the overall region model passed the 1% significance test. It shows that there are large differences in R&D investment between the regions, so the technological advancement effect and application value brought on by R&D investment are different. Additionally, the practicability of R&D investment in the downstream region and midstream region is stronger than that in the upstream region. Therefore, there is still a lot of room for improvement in the upstream region; its technological advancement has greater elasticity, and its promoting effect on TFP is also stronger. This provides an idea for the western region to achieve leapfrog development, that is, to strengthen R&D investment to promote technological advancement.

#### 5.3.4. Industrial Structure

The upgrading of the industrial structure is an inevitable path in the progress of high-quality economics. From the regression results, the *lnIS* coefficients of the overall region, upstream region and midstream region of the Yangtze River Economic Belt are −0.987, −20.38 and −5.982, respectively. Additionally, the *lnIS* coefficient of the downstream region is 2.971. Statistically, the result of *lnIS* in the upstream region and midstream region model passed the 1% significance test. Although the results are somewhat different from theoretical expectations, the four provinces and cities in the downstream region give a strong signal that in order to achieve high-quality economic development, we must complete the upgrading of the industrial structure. Only through this can the TFP be effectively improved on the industrial level. Similarly, the upstream region and midstream region are still in the stage of undertaking industry, but the industries they undertake are facing greater challenges to effectively promote the growth of TFP.

#### 5.3.5. Government Expenditure

In a market economy, the means of government regulation of the economy is no longer dominated by administrative intervention, but by affecting market prices or investment returns to improve the industrial structure and their geographic distribution. Therefore, government expenditure can be regarded as the main means of regulating the market. From the regression results, the *lnM* coefficients of the overall region, upstream region and midstream region of the Yangtze River Economic Belt are −1.709, −11.75 and −15.03, respectively, and the *lnM* coefficient of the downstream region is 5.717. Statistically, the result of *lnM* in the upstream region, midstream region and downstream region model passed the 1% significance test. Therefore, the promotion effect of government expenditure in the downstream region on TFP is more obvious. In fact, the downstream region of the Yangtze River Delta region, as a key development region of the country, has strong policy support, and the business environment and social atmosphere are more perfect. There is still a gap in policy support and effect between the upstream region and midstream region.

#### 5.3.6. Human Capital

Human capital is the key factor of enterprises’ or regions’ competition. From the regression results, the *lnHC* coefficients of the overall region and upstream region of the Yangtze River Economic Belt are −3.209 and −7.760, respectively, and the signs are inconsistent with theoretical expectations. The *lnHC* coefficients of the midstream region and downstream region are 11.03 and 2.482, respectively, and the signs are consistent with theoretical expectations. Statistically, the result of *lnHC* in the upstream region, midstream region and downstream region model passed the 1% significance test. It shows that human capital can promote the growth of TFP in the following order: the midstream region is the strongest, then the downstream region, while the upstream region has an inhibitory effect. This difference is basically consistent with the reality of the regional development gap. The attractiveness to talent just shows in the order of the downstream region, midstream region and upstream region from strong to weak, which has just been verified in the growth of regional TFP.

## 6. Conclusions

In summary, the top-down economic development differences in the Yangtze River Economic Belt have been fully explained by total factor productivity, and the factors that lead to and affect this difference have also been empirically tested. Therefore, the rise of the ‘Yangtze River Delta regional integration’ as a national strategy has brought unprecedented opportunities for the high-quality and coordinated development of the Yangtze River Economic Belt. In particular, the upstream and midstream of the Yangtze River Economic Belt will break through the constraints of various factors and achieve leapfrog high-quality development. The following aspects still need to be carried out in this process.

### 6.1. Comprehensively Improve the Overall Level of Opening up to the Outside World of the Yangtze River Economic Belt

China’s Yangtze River Delta region’s share of overall national GDP increased from 24.1 percent in 2018 to 24.5 percent in 2021. The region has generally become a strong and active growth engine to China’s economic development. In the expansion of opening up, the Yangtze River Delta region should give full play to the demonstration and driving role of the international economic growth pole. It should not only further expand the open field of the region and improve the level of opening up, but also radiate the super-strong factor agglomeration ability, complete industrial chain and high-end advantages of the value chain to the midstream region and upstream region of the Yangtze River Economic Belt. At the same time, for the midstream region and upstream region of the Yangtze River Economic Belt, which is relatively backward in development, in the process of expanding internal and external opening up, they should not only increase production capacity, but also improve quality, especially focusing on leading industries, emerging materials, advanced manufacturing and related pillar industries, so as to realize the leapfrog high-quality development of the region.

### 6.2. Strengthening R&D Investment, Cross-Regional Cooperation in Scientific Research and Application Transformation

Scientific research and innovation cycle are long and return efficiency is slow, but R&D investment is the most significant to improve TFP. Therefore, provinces and cities in the Yangtze River Economic Belt should strengthen R&D investment, effectively break the constraints of factor endowment, and provide original impetus for high-quality economic development. At the same time, there is a large gap between the stock and increment of R&D investment in the upstream region, midstream region and downstream region of the Yangtze River Economic Belt, and the innovation factors were realized are not equal. Strengthening cross-regional cooperation in scientific research and improving the overall region application transformation capability are the top priorities of 11 provinces and cities along the route. At present, the four provinces and cities in the downstream region are promoting the construction of regional integration, and the barriers in scientific research system are expected to be eliminated. The seven provinces and cities in upstream region and midstream region should strengthen close cooperation with downstream region in scientific research.

### 6.3. The Industrial Transfer and Structural Upgrading of the Yangtze River Economic Belt Should Be Coordinated as a Whole

The upstream region, midstream region and downstream region of the Yangtze River Economic Belt are in different stages of development. Therefore, the development gap between regions and the difference in the growth space of TFP have brought the possibility of industrial transfer between regions. In the process of cultivating and building world-class industrial clusters, the green and coordinated development of the natural-economic-social system of the basin is continuously promoted [33]. In the short term, the industries in the industrial chain of the downstream region that have little effect on promoting its TFP can be gradually transferred to the midstream region and upstream region. In the long run, the overall industrial layout of the upstream region, midstream region and downstream region of the Yangtze River Economic Belt should be well coordinated, not just limited to the transfer of industries and the replacement of space. The coordinated development of different regions will release more development opportunities for China in the future [34]. Not only should the integrity of the industrial chain and the high-end value chain be fully considered, but also more attention should be paid to the innovation and sustainability of the industrial chain so that it can effectively realize the green, coordinated and innovative development of the industries in the Yangtze River Economic Belt.

### 6.4. Playing the Guiding Role of Government Expenditure Actively and Effectively to Stabilize the Market Demand and Investment Confidence

Although the market plays a decisive role in resource allocation, the government’s active and effective macro-control can make up for the insufficiency of the market mechanism. In particular, the world economy is currently facing the impact of the COVID-19 pandemic, coupled with the large fluctuations in recent international oil prices and related resource prices, the downward trend of the world economy is difficult to reverse, the overall demand may further shrink, and the economic growth of various countries in the world are facing new uncertainties. Therefore, playing the guiding role of government expenditure well, especially in the field of investment, can effectively stabilize the market demand and the investment confidence of market entities in the Yangtze River Economic Belt, thereby improving TFP.

### 6.5. Promote the Free Flow of Human Capital in the Yangtze River Economic Belt

Human capital is a key element for high-quality economic development. The fundamental driving force for the flow of factors is to realize and increase the factors’ market value. Only by creating and realizing value proliferation can the continuous circulation of factors be promoted. Similarly, accelerating the free flow of human capital between provinces and cities in the Yangtze River Economic Belt and continuously realizing the value of human capital elements is the fundamental guarantee for improving the overall TFP of the Yangtze River Economic Belt and improving its status in the global value chain. The free flow of human capital factors, especially the two-way flow between relatively underdeveloped regions and developed regions, can effectively narrow the gap between the stock and increment of human capital between regions, reduce the gap in TFP, and achieve high-quality economic development.

## Figures and Tables

**Table 1:** Influencing factors of TFP selected and their relationship with TFP.

Variable	Meaning	Measurement Methods	Relationship with TFP
*TRADE*	International trade dependence	The ratio of total imports and exports to GDP	positive correlation
*FDI*	FDI dependence	The ratio of FDI to GDP	positive correlation
*TECH*	R&D expenditure	R&D spending as a percentage of GDP	positive correlation
*IS*	Industrial structure	The added value of the tertiary industry as a percentage of GDP	positive correlation
*M*	Government expenditure	General budget spending as a share of GDP	positive correlation
*HC*	Human capital	Total high school and higher education graduates	positive correlation

**Table 2:** The TFP growth rate (*tfp*) of the Yangtze River Economic Belt from overall region, upstream region, midstream region and downstream region from 2007 to 2018 (unit: %).

Year	Overall Region	Upstream Region	Midstream Region	Downstream Region
2007	10.86	11.95	12.66	9.54
2008	8.60	10.81	11.10	6.69
2009	8.07	8.48	9.96	7.08
2010	8.81	7.56	10.64	8.52
2011	6.41	8.02	8.37	5.06
2012	3.37	5.47	4.10	2.22
2013	3.08	3.03	2.76	3.26
2014	2.70	1.53	2.93	3.14
2015	1.79	0.46	1.58	2.48
2016	3.44	1.70	2.93	4.54
2017	0.74	0.83	-0.94	1.55
2018	0.83	−0.60	2.01	0.96

Data source: National Bureau of Statistics, statistical yearbooks of provinces and cities from 2007 to 2019. Note: The 11 provinces and cities in the Yangtze River Economic Belt are Shanghai, Jiangsu, Zhejiang, Anhui, Hubei, Hunan, Jiangxi, Sichuan, Yunnan, Guizhou and Chongqing. The downstream region includes Shanghai, Jiangsu, Zhejiang and Anhui. The midstream region includes Hubei, Hunan and Jiangxi. The upstream region includes Sichuan, Yunnan, Guizhou and Chongqing.

**Table 3:** Summary statistics of *tfp* in 4 regions.

Region	Mean	Maximum	Minimum	Std. Dev.	Observations
Overall Region	4.890	10.862	0.738	3.478	12
Upstream region	4.937	11.952	−0.600	4.33	12
Midstream region	5.676	12.682	−0.940	4.559	12
Downstream region	4.586	9.540	0.958	2.817	12

**Table 4:** Regression results of *tfp* influencing factors of the overall regions, upstream regions, midstream regions and downstream regions in the Yangtze River Economic Belt.

Explanatory Variables	Overall Region		Upstream Region		Midstream Region		Downstream Region	
*lnTRADE*	−1.573	***	−2.043		1.869	***	5.252	***
	(0.341)		(1.510)		(0.701)		(0.606)	
*lnFDI*	0.272		−1.721	***	5.498	***	1.337	
	(0.583)		(0.500)		(2.100)		(1.029)	
*lnTECH*	2.170		5.809	***	−7.209	***	−15.99	***
	(2.333)		(2.165)		(0.280)		(2.299)	
*lnIS*	−0.987		−20.38	***	−5.982	***	2.971	
	(2.865)		(2.843)		(1.931)		(2.439)	
*lnM*	−1.709		−11.75	***	−15.03	***	5.717	***
	(2.020)		(3.779)		(2.615)		(1.694)	
*lnHC*	−3.209		−7.760	***	11.03	***	2.482	***
	(1.987)		(2.813)		(2.640)		(0.876)	
*tfp (L1)*	0.741 (0.123)	***						
*Constant*	24.38		155.3	***	19.78	***	−40.59	**
	(21.05)		(15.25)		(6.620)		(17.56)	
Observations	121		48		36		48	
R-squared	0.839		0.705		0.879		0.855	
Number of Pro	11		4		3		4	

Note: Data in parentheses are standard deviations. Symbol meaning: *** *p* < 0.01, ** *p* < 0.05.

## Appendix A

**Table A1:** The abbreviations of key terms.

Key Term	Abbreviations
total factor productivity	TFP
the Communist Party of China	CPC
research and development	R&D
foreign direct investment	FDI
gross domestic product	GDP
TFP growth rate	*tfp*
the actual output of the base period in 2005	*Y_t_*
the capital stock	*K_t_*
the labor input	*L_t_*
the technological advancement factor	*A_t_*
the capital output elasticity coefficient	*α*
the labor output elasticity coefficient	*β*
the error term	*u_t_*
the real GDP growth rate	*y*
International trade dependence	*TRADE*
FDI dependence	*FDI*
R&D expenditure	*TECH*
Industrial structure	*IS*
Government expenditure	*M*
Human capital	*HC*
